# FMDV 3A cooperates with PDCD10 to promote FMDV replication by inhibiting VISA-mediated innate immunity

**DOI:** 10.1128/jvi.00657-25

**Published:** 2025-11-26

**Authors:** Qian Li, XiaoFeng Nian, XiaoFen Shang, ZongBo Zeng, ZhiKuan Luo, Bo Du, MeiHua Ma, ZiXiang Zhu, Fan Yang, JingJing Pei, WeiJun Cao, HongBin Yan, Li Li, YiGang Xu, XuSheng Ma, HaiXue Zheng

**Affiliations:** 1 China-Malaysia National Joint Laboratory, Biomedical Research Center, Life Science and Engineering College, Northwest Minzu University 66293, Lanzhou, China; 2 State Key Laboratory for Animal Disease Control and Prevention, Gansu Province Research Center for Basic Disciplines of Pathogen Biology, African Swine Fever Regional Laboratory of China, College of Veterinary Medicine, Lanzhou University, Lanzhou Veterinary Research Institute, Chinese Academy of Agricultural Sciences 111658 , Lanzhou, China; 3 College of Veterinary Medicine, Northeast Agricultural University 12430, Harbin, China; 4 College of Veterinary Medicine, Gansu Agricultural University 739715, Lanzhou, China; 5 Key Laboratory of Applied Technology on Green-Eco-Healthy Animal Husbandry of Zhejiang Province, College of Animal Science & Technology, College of Veterinary Medicine, Zhejiang A&F University 722545, Hangzhou, China; Loyola University Chicago - Health Sciences Campus, Maywood, Illinois, USA

**Keywords:** foot-and-mouth disease virus, 3A, PDCD10, innate immunity, VISA, interferons, virus replication

## Abstract

RIG-I like receptors (RLRs) recognize RNA viruses and induce an innate immune response. Although many host factors strictly regulate the signal transduction of the RLR pathway, the mechanisms remain unclear. In the present study, we demonstrated that virus infection slightly increased the expression of programmed cell death protein 10 (PDCD10). PDCD10 overexpression inhibited interferon beta (IFN-β) promoter activation after Sendai virus (SeV) infection. Moreover, PDCD10 negatively regulated RNA virus-induced IFN-β secretion. These effects were reversed following PDCD10 gene knockout. PDCD10 also interacted with virus-induced signaling adaptor (VISA) and disrupted the formation of the VISA-IRF3 complex to inhibit IFN-β production. Additionally, PDCD10 promoted foot-and-mouth disease virus (FMDV) replication by inhibiting IFN-β production. FMDV is the causative pathogen of foot-and-mouth disease, one of the most destructive and contagious animal diseases in the world. The FMDV 3A protein plays important roles in viral replication, host tropism, and immune regulation. Our experimental results also showed that full-length 3A cooperated with PDCD10 to inhibit IFN-β production by promoting the binding of PDCD10 to VISA. Collectively, the study findings revealed that PDCD10, as a new negative regulator, cooperated with 3A to inhibit viral-induced IFN-β production.

**IMPORTANCE:**

Foot-and-mouth disease virus (FMDV) is a pathogen that causes a highly contagious and destructive foot-and-mouth disease in animals with cloven hooves. Although the 3A protein of FMDV is involved in viral replication and host tropism, its function remains unclear. PDCD10 plays critical roles in normal cardiovascular development, cell proliferation, and normal structure and assembly of the Golgi complex. The present study showed that PDCD10 expression was slightly increased by virus infection, while PDCD10 promoted FMDV replication. Our results also demonstrated that PDCD10 inhibited Sendai virus-induced interferon beta (IFN-β) production through interaction with virus-induced signaling adaptor (VISA). PDCD10 also disrupted VISA-IRF3 complex formation to impair IFN-β production induced by RNA virus. The FMDV 3A protein bound with PDCD10 to synergistically promote FMDV replication. This study helped to reveal the potential mechanism of FMDV 3A protein and PDCD10 impact on viral replication.

## INTRODUCTION

Foot-and-mouth disease virus (FMDV) belongs to the genus *Aphthovirus* of the Picornaviridae family, and it causes a highly contagious and severe disease with vesicle formation and erosions in the epithelium of animals with cloven hooves (1). The single positive-stranded RNA genome of FMDV encodes four structural proteins (VP1, VP2, VP3, and VP4) and eight nonstructural proteins (L^pro^, 2A, 2B, 2C, 3A, 3B, 3C, and 3D) (2). The 3A protein of FMDV anchors on the intracellular membrane with its hydrophobic motif and mediates the localization of the FMDV replication complex on the cell membrane. A previous study demonstrated that 3A interacts with vimentin to negatively modulate FMDV replication (3). The 3A also combines with DDX56 to inhibit IRF3 phosphorylation to enhance FMDV replication (4). We previously observed that 3A suppresses the ANXA1 effect to positively promote FMDV replication (5). Sar1 and Sec12 were hijacked by FMDV 3A for endoplasmic reticulum remodeling in a COPII-independent manner (6). Although the 3A protein is one of the critical components of FMDV replication, its underlying function remains unclear.

PDCD10 (also named CCM3 or TFAR-15) is a conserved gene from nematode to human; it is also identified as the third causative gene of CCM and is an apoptosis-related gene (7, 8). PDCD10 plays essential roles in cell proliferation, protein synthesis, developmental disorders, and other diseases (9). The well characterization of PDCD10 is based on its function as a component of the STRIPAK (striatin-interacting phosphatase and kinase) complex that interacts with STK24, MST4 (serine/threonine-protein kinase 26), and STK25 GCKIII kinase subfamily (10–13). PDCD10 overexpression also decreased NF-κB p65 level and the level of inflammatory factors such as TNF-α and IL-1β (14).

Melanoma differentiation-associated protein 5 (MDA5) and retinoic acid-inducible gene I (RIG-I) are RNA sensors that induce antiviral immune response. After the viral RNA genome is recognized by MDA5 or RIG-I, both sensors use their N-terminal Caspase recruitment domain (CARD) to interact with virus-induced signaling adaptor (VISA, also named mitochondrial antiviral-signaling protein, [MAVS]), which recruits signaling kinases such as TBK1, IκB kinase (IKK) family kinases, to activate IRF3 or NF-κB. The activated NF-κB and IRF3 can translocate into the nucleus and interact with specific promoter regions to initiate the type I interferon (IFN) production, which induces the expression of hundreds of IFN-stimulated genes (ISGs), leading to the establishment of the antiviral state (15, 16).

Multiple host factors tightly control signaling by RIG-I-like receptors (RLRs) to prevent excessive inflammatory responses. For instance, tripartite motif protein 25 (TRIM25) interacts with RIG-I to facilitate the K172 site ubiquitination of RIG-I CARD 2 (17). A previous study showed that the RIG-I CARD domain was phosphorylated by protein kinase C α (PKC-α) and PKC-β to suppress RIG-I–TRIM25 interaction, thus inhibiting RLRs-mediated antiviral responses (18). Another study demonstrated that Atg5–Atg12 suppressed RIG-I–MAVS interaction to modulate RIG-I signaling transduction (19). Restricting the regulation of RLR-mediated antiviral effects is crucial for the host immune response; however, the mechanism of its regulation is not fully understood.

In the present study, we used the luciferase reporter system to screen for host proteins that regulate type I IFN and found that PDCD10 was a candidate for suppressing viral RNA-induced IFN-β production. We determined that PDCD10 overexpression decreased IFN-β promoter activity to inhibit the expression of ISGs. These results were reversed after PDCD10 knockdown or knockout during Sendai virus (SeV) or vesicular stomatitis virus (VSV) infection. The underlying mechanism was that PDCD10 disrupted the formation of a complex between VISA and IRF3 during viral infection. FMDV titer was increased after PDCD10 overexpression. During the viral infection process, FMDV also uses PDCD10 to further inhibit IFNβ production and promote FMDV replication. The FMDV intact 3A protein cooperated with PDCD10 to inhibit IFN-β production, while 3A also promoted the binding of PDCD10 to VISA. Taken together, our data suggested that PDCD10 inhibited VISA-mediated innate immune responses, while FMDV 3A cooperated with PDCD10 to promote FMDV replication. The present study expanded our knowledge of novel functions of the 3A protein and PDCD10 in the regulation of immune responses against FMDV.

## MATERIALS AND METHODS

### Cells

Porcine kidney 15 (PK15), baby hamster kidney 21 (BHK21), human embryonic kidney 293, Instituto Biologico-Rim Suino-2 (IBSR-2), and Henrietta Lacks (HeLa) cell lines from ATCC were stored in our lab, while PDCD10-knockout HeLa cells were constructed by our lab. Porcine alveolar macrophages (PAMs) were collected from the lung tissue of 3-month-old pigs. PK15, IBSR-2, and BHK21 cells were maintained in modified Eagle’s medium (MEM; Gibco); HeLa and HEK293 cells were maintained in Dulbecco’s modified Eagle’s medium (DMEM; Gibco). DMEM and MEM were supplemented with 10% fetal bovine serum, 100 U/mL penicillin, 100  µg/mL streptomycin, and the cells were cultured at 37°C under 5% CO_2_ atmosphere. The cells were free of mycoplasma contamination.

### Viruses

FMDV (FMDV/O/BY/CHA/2010), SeV, Seneca Valley virus (SVA), enterovirus 71 (EV71), and VSV (VSV-GFP/VSV-RFP) were maintained in our lab. African swine fever virus (ASFV) was stored in our institute. VSV-GFP replication was performed in HEK293 cells. SeV was propagated in embryonated chicken eggs. FMDV and SVA replication or titration was performed in BHK21 cells or IBSR-2.

### Reagents

Monoclonal antibodies against PDCD10 (SAB1412804), Flag (F2555), HA (H3663), and myc (SAB2702192) were purchased from Sigma-Aldrich (Merck Ltd [China], Beijing, China). Primary antibodies against IRF3 (#11904), TRAF6 (#67591), TRAF3 (#33640), TBK1 (#38066), p-IRF3 (#37829), p-TBK1 (#5483), and β-actin (#4970) were purchased from Cell Signaling Technology, Co. Inc. Lipofectamine 2000/3000 was obtained from Invivogene Biotech Co., Ltd., and TRIzol was obtained from Invitrogen (Thermo Fisher Scientific [China] Co., Ltd., China). PDCD10 purified protein (HY-P71190) and VISA (Ag5949) were ordered from MedChemexpress (MCE, USA) and Proteintech (Proteintech Group, Inc., USA). Z-VAD-FMK and Dinaciclib were purchased from MedChemExpress (MCE, USA).

### Plasmid construction and transfection

The DNA fragment of PDCD10 was generated by PCR amplification of the total RNA of PK15 cells. The plasmids containing porcine VISA, STAT1, JAK1, or TBK1 were stored in our laboratory. The plasmids of TRAF3, TRAF6, IRF3, and IRF3-5D (Hongbing Shu’ lab) were kept in our laboratory. The plasmid of the 3A gene from FMDV/O/BY/CHA/2010 was also stored in our lab. All the expression plasmids were constructed into the plasmid pcDNA3.1 and sequenced in the Beijing Genomics Institute. Lipofectamine 2000 or 3000 was used for transfection according to the manufacturer’s instructions.

### Luciferase reporter assay

HEK293 or PK-15 cells (1 × 10^5^) were cultured in 48-well plates for 24 h. 20 ng of pRL-TK reporter plasmid and 200 ng of IFN-β or interferon-stimulated response element (ISRE) reporter plasmid were transfected into HEK293 cells (IFN-β, ISRE, and pRL-TK reporter plasmids as gifts were provided by Shu Hong Bing’s Lab, Wuhan University, China). After 24 h post-transfection, the cells were either not treated or infected with SeV/FMDV for 12 h. The luciferase activity was measured with the Dual-Luciferase Reporter Assay Kit (Promega, USA) according to the manufacturer’s protocol.

### Lentivirus production and infection

The target sequence of PDCD10 short hairpin RNA (shRNA) was 5ʹ-CAGGATGTTGAATGGGATTAT-3ʹ. Annealed PDCD10 shRNA-synthesized cDNA fragments or a negative control shRNA were digested with *EcoRI* and *BamHI* and cloned into the pLVX shRNA expression empty vector (HANBIO, Shanghai, China). The shPDCD10 lenti-vector, psPAX2, and pMD2.G were transfected into HEK293 cells. Culture supernatants were harvested at 48 and 72 h; the culture medium was filtered with a 0.45 µm filter and centrifuged at 72,000 × *g* for 120 min at 4°C. The shRNA knockdown efficiency was assessed by qPCR and western blot analysis.

### RNA extraction and RT-PCR

Total RNA was extracted using TRIzol reagent (Invitrogen, USA). M-MLV reverse transcriptase (Promega, USA) and random hexamer primers (Takara, Japan) were used to prepare cDNA. The generated cDNA was used as a template for FMDV RNA and cellular mRNA host expression. Real-time quantitative PCR (RT-PCR) was performed to measure the abundance of different mRNAs using Mx3005P qPCR (Agilent Technologies, USA) and SYBR Premix ExTaq reagents (TaKaRa, Japan). The data were normalized to GAPDH expression. The 2^−ΔΔCt^ method was used to calculate the relative expression of mRNA. RT-qPCR primers are listed in Table 1.

**TABLE 1:** Primers used for qPCR^*a*^

Primer	Sequence
pIFN-β Forward	5′TGCAACCACCACAATTCC3′
pIFN-β Reverse	5′CTGAGAATGCCGAAGATCTG3′
pPDCD10 Forward	5′ GGGGAATTGATCCGGGAGTT3′
pPDCD10 Reverse	5′ ACATATACTGTCCGGCAACTGA3′
pISG56 Forward	5′ACGTAACTGAAAATCCACAAGA3′
pISG56 Reverse	5′TGCTCCAGACTATCCTTGACCT3′
pISG20 Forward	5′CTCCTGCACAAGAGCATCCA3′
pISG20 Reverse	5′CATCGTTGCCTTCGCATCT3′
pGAPDH Forward	5′ACATGGCCTCCAAGGAGTAAGA3′
pGAPDH Reverse	5′GATCGAGTTGGGGCTGTGACT3′
FMDV Forward	5′ACTGGGTTTTACAAACCTGTGA3′
FMDV Reverse	5′GCGAGTCCTGCCACGGA3′
hIFN-β Forward	5′TTGTTGAGAACCTCCTGGCT3′
hIFN-β Reverse	5′TGACTATGGTCCAGGCACAG3′
hPDCD10 Forward	5′TGGCAGCTGATGATGTAGAAG3′
hPDCD10 Reverse	5′TCGTGCCTTTTCGTTTAGGT3′
hISG56 Forward	5′GCCTTGCTGAAGTGTGGAGGAA3′
hISG56 Reverse	5′ATCCAGGCGATAGGCAGAGATC3′
hISG54 Forward	5′CACCTCTGGACTGGCAATAGC3′
hISG54 Reverse	5′GTCAGGATTCAGCCGAATGG3′
hGAPDH Forward	5′GAGTCAACGGATTTGGTCGT3′
hGAPDH Reverse	5′GACAAGCTTCCCGTTCTCAG3′
SeV Forward	5′TGTTATCGGATTCCTCGACGCAGTC3′
SeV Reverse	5′TACTCTCCTCACCTGATCGATTATC3′
VSV Forward	5′CTGGGTTAGCTTTGGAAGAA3′
VSV Reverse	5′CCAGAAGTGAAAGCTGGA3′
SVA Forward	5′AACCGGCTGTGTTTGCTAGAG3′
SVA Reverse	5′GAACTCGCAGACCACACCAA3′

^*a*^ p, porcine; h, human.

### Co-immunoprecipitation assay

The indicated plasmids were transfected into HEK293 cells, and the cells were cultured in 10 cm^2^ plates. After 24 h, the cells were collected and lysed in lysis buffer (20 mM Tris [pH 7.5], 150 mM NaCl, 1% Triton X-100, 1 mM EDTA, 10 mg/mL aprotinin, 10 mg/mL leupeptin, and 1 mM PMSF). Lysates were incubated with 1 µg of primary antibody or control IgG and 60 µL of G-Sepharose (GE Healthcare, USA) for 6 h. Sepharose beads were washed three times with 1 mL of lysis buffer containing 500 mM NaCl. Immunoblotting was performed to analyze the precipitates.

### Pull-down assay

Purified VISA and PDCD10 were dissolved in cell lysis buffer and then mixed together, and anti-VISA antibody was added and incubated overnight at 4°C. Protein A/G agarose beads were added into the mixture and incubated for 3 h, then centrifuged at 12,000 rpm for 30 sec, the beads were washed with lysis buffer five times (each for 5 min), and then loading buffer was added, boiled for 10 min, and the samples were detected by western blotting with indicated antibody.

### Western blotting

Cell samples were boiled after lysis with a lysis buffer for 30 min. Cell lysates were subjected to SDS-PAGE (Bio-Rad), and the separated proteins were transferred onto PVDF membranes (Millipore). The membranes were blocked in TBST (Tris-buffered saline with 0.1% Tween 20) with 5% fat-free milk for 1 h at room temperature and then incubated with primary antibodies in 5% BSA overnight at 4°C. The membranes were then washed again with TBST five times and finally incubated with horseradish peroxidase-conjugated secondary antibodies in 5% BSA at room temperature for 1 h. The membranes were then imaged by a ChemiDoc MP Imaging System (Bio-Rad, USA).

### Immunofluorescence microscopy

The cells added with Mito-Tracker (100 mM) were incubated at 37°C for 30 min. The indicated cells were washed with cold phosphate-buffered saline (PBS) three times and fixed with 4% paraformaldehyde at room temperature for 15 min. HeLa cells were permeabilized with 0.1% Triton X-100 at room temperature for 5 min and blocked with 10% goat serum and 0.2% Tween-20 in PBS (PBST). Primary and secondary antibodies were solubilized in 10% goat serum in PBST. The cells were incubated with primary antibodies overnight at 4°C. Subsequently, the cells were washed five times and incubated with secondary antibodies for 1 h. The slides were then stained with DAPI for 15 min and imaged with a laser-scanning confocal microscope (LSCM; SP8, Leica, Solms, Germany).

### Statistical analysis

All tests were reproducible, and similar findings were confirmed by repetition at least three times. Sample variation was determined using Tukey’s post hoc test and analyzed by one-way ANOVA or two-way ANOVA. Means are represented with histograms, with error bars representing the standard error of the mean (s.e.m.). The *P* values < 0.05 were considered statistically significant.

## RESULTS

### PDCD10 expression level was increased after viral infection

**Fig 1:**
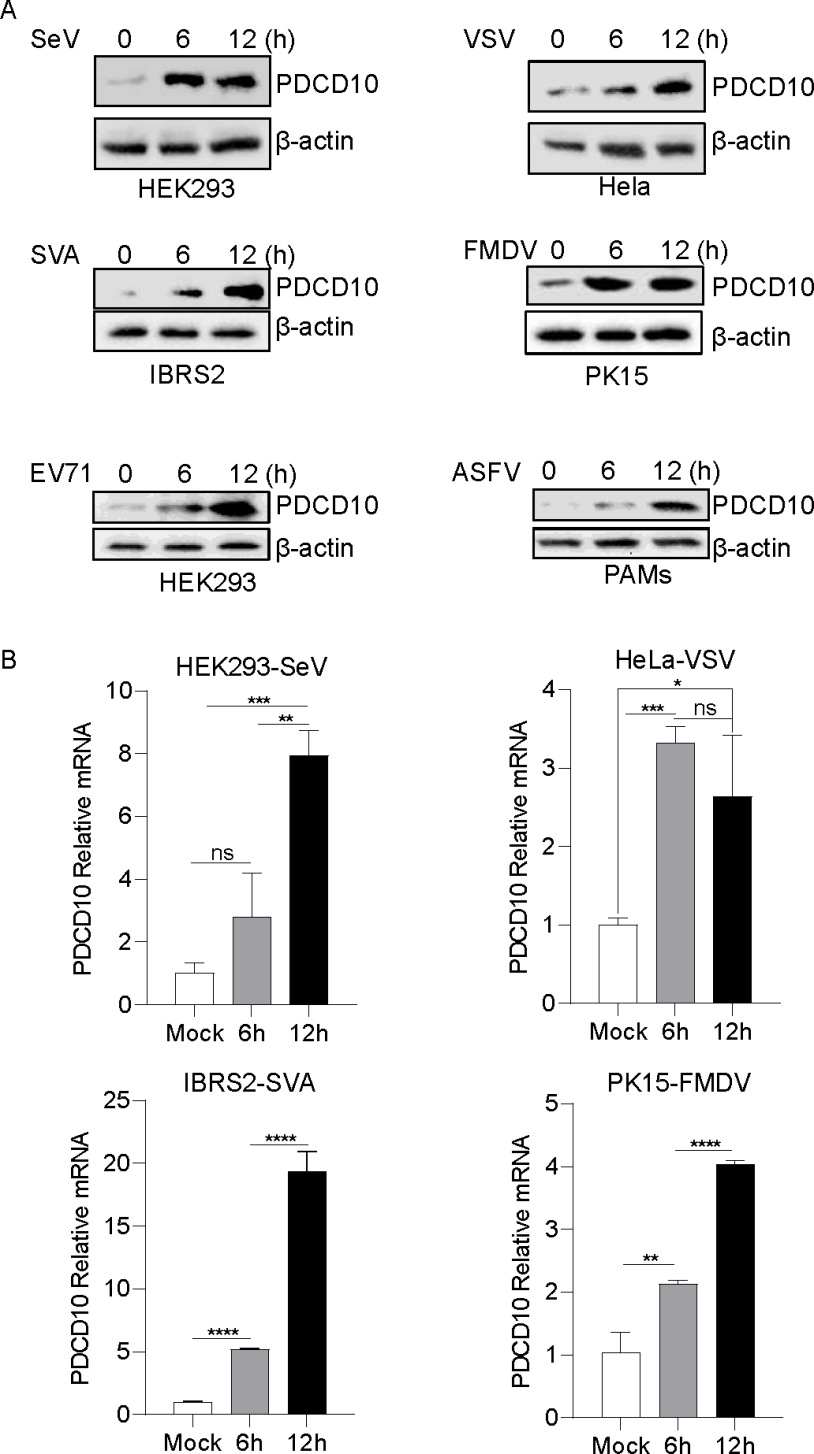
Viral infection increased PDCD10 expression. (**A**) HeLa, IBRS2, HEK293, PK15 cells, or PAMs were infected with SeV, VSV, FMDV, SVA, or ASFV (multiplicity of infection [MOI] = 5) at the indicated time points. Cell lysates were subjected to a western blotting assay. (**B**) HeLa, IBRS2, HEK293, or PK15 cells were infected with SeV, VSV, FMDV, or SVA (MOI = 5) at the indicated time points. Cellular RNA was extracted and reverse transcribed into cDNA. Compared to the control, relative mRNA levels of PDCD10 in virus-infected cells were detected by qPCR. Data are representative of three independent experiments. The data are expressed as mean ± s.e.m; *P < 0.05, **P < 0.01, ***P < 0.001, ****P < 0.0001 (two-way ANOVA, GraphPad Prism 8.3.0).

To determine whether infection with viruses affects PDCD10 expression, the mRNA and protein expression levels of PDCD10 were assessed after viral infection. As shown in Fig. 1A, PDCD10 protein expression was slightly increased in SeV-infected HEK293 cells and VSV-infected HeLa cells and significantly increased in SVA-infected IBRS2 cells and FMDV-infected PK-15 cells. To determine whether *picornaviridae* of the same family as FMDV also affect PDCD10 expression, HEK293 cells were infected with EV71, we found that EV71 promotes PDCD10 protein expression (Fig. 1A). Furthermore, to confirm whether the conclusion that viruses promote PDCD10 expression extends to DNA viruses, ASFV was used to infect PDCD10, and the results showed that ASFV also increased the PDCD10 protein level (Fig. 1A). Moreover, as shown in Fig. 1B, the PDCD10 mRNA levels in HEK293, IBRS2, PK-15, or HeLa cells were increased after SeV, VSV, SVA, or FMDV infection. These results suggested that PDCD10 expression was associated with viral infection.

### PDCD10 as a new candidate to inhibit the IFN-β production

**Fig 2:**
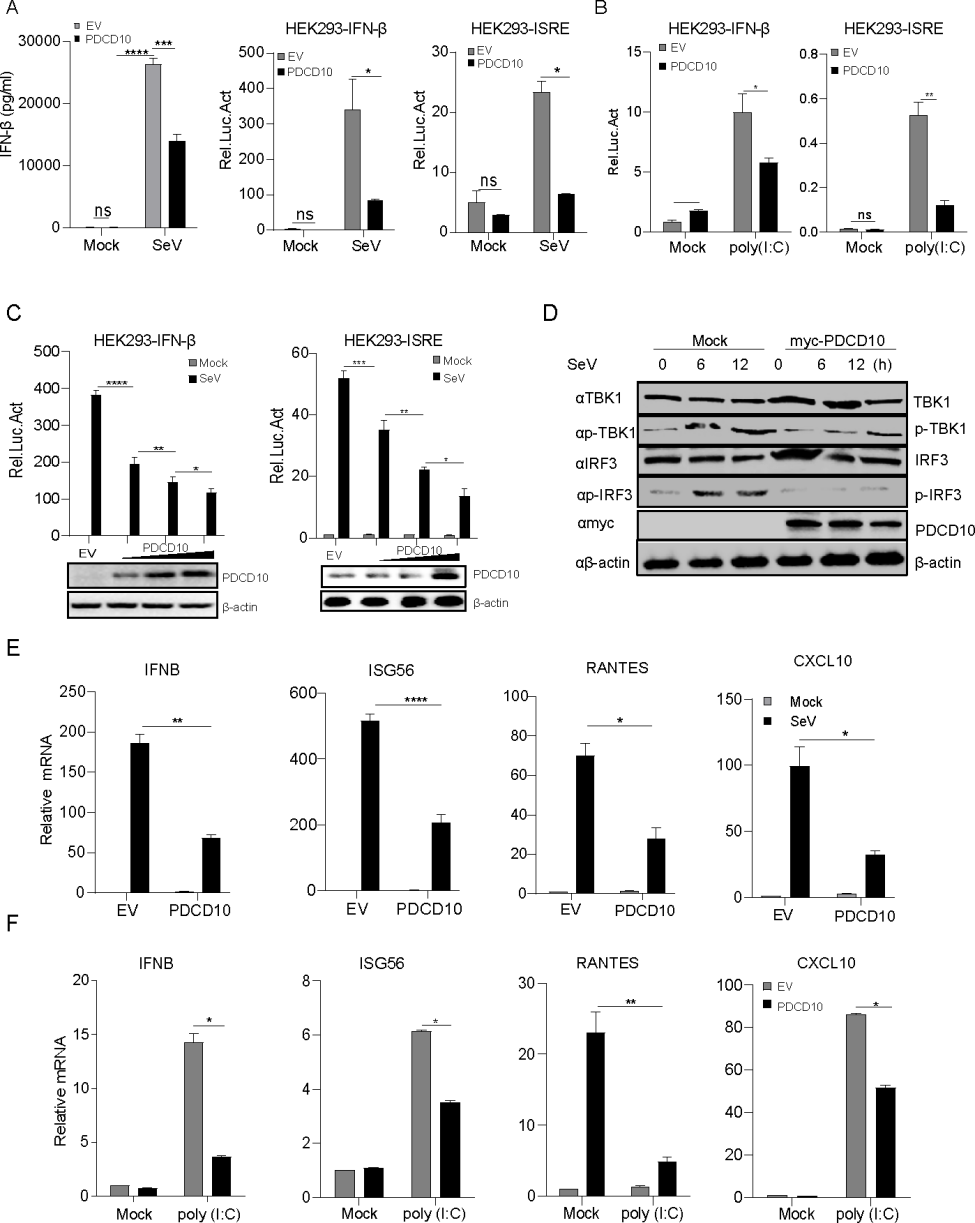
PDCD10 suppressed SeV-induced IFN-I activation. (**A**) Plasmids of PDCD10 were transfected into HEK293 cells, after 24 h, the cells were infected with SeV for 12 h. Then, IFN-β was assayed with ELISA; plasmids of PDCD10 (300 ng) and pRL-TK (20 ng) together with pIFN-β-luc or pISRE-luc (200 ng) were transfected into HEK293 cells. After 24 h, the cells were infected with SeV, and after 12 h, cell lysates were analyzed for the relative intensity of luciferase. (**B**) Plasmids containing PDCD10 and pRL-TK (20 ng) together with pIFN-β-luc or pISRE-luc (200 ng) were transfected into HEK293 cells. After 24 h, the cells were transfected with poly (I: C), and after 24 h, cell lysates were analyzed for the relative intensity of luciferase. (**C**) Plasmids containing PDCD10 (indicated dose) and pRL-TK (20 ng) together with pIFN-β-luc or pISRE-luc (200 ng) were transfected into HEK293 cells. After 24 h, the cells were infected with SeV for 12 h, and cell lysates were analyzed for the relative intensity of luciferase. (**D**) PDCD10 (2 µg) was transfected into HEK293 cells. After 24 h, the cells were infected with SeV at the indicated time points, and cell lysates were subjected to Western blotting. (**E**) PDCD10 plasmid (1 µg) was transfected into HEK293 cells. After 24 h, the cells were infected with SeV for 12 h, cell RNA was extracted and reverse transcribed into cDNA. Relative mRNA levels were detected by qPCR. (**F**) PDCD10 plasmid (1 µg) was transfected into HEK293 cells. After 24 h, the cells were transfected with poly (I: C). After 24 h, cellular RNA was extracted and reverse transcribed into cDNA. Relative mRNA levels were detected by qPCR. Data are representative of three independent experiments. The data shown are the mean ± s.e.m; *P < 0.05, **P < 0.01, ***P < 0.001, ****P < 0.0001 (two-way ANOVA, GraphPad Prism 8.3.0).

IFN-β production is induced through the RLRs signaling pathway during RNA virus infection. To elucidate the regulators that affect IFN-I production, we assessed the IFN-β secretion with enzyme-linked immunosorbent assay (ELISA), and we found that PDCD10 inhibits IFN-β secretion during SeV or VSV infection (Fig. 2A; Fig. S1A). Meanwhile, we performed a luciferase assay to accurately identify the proteins that inhibit the IFN-β promoter. The results of the luciferase assay revealed that PDCD10 could inhibit SeV-induced IFN-β and ISRE promoter activation (Fig. 2A). Poly (I: C) could simulate the RNA virus genome to induce the IFN-I production. We observed that PDCD10 overexpression inhibited IFN-β and ISRE activation after poly (I: C) transfection (Fig. 2B). IFN-β promoter activation was inhibited by PDCD10 in a dose-dependent manner in HEK293 cells after virus infection (Fig. 2C). Phosphorylated IRF3 translocates into the nucleus to induce IFN-β production. We found that PDCD10 overexpression suppressed SeV-induced phosphorylation of IRF3 (Fig. 2D). The expression of ISGs is associated with IFN-I production. PDCD10 was transfected into HEK293 cells, and after 24 h, the cells were infected with SeV or VSV for 12 h. As shown in Fig. 2E and Fig. S1B, PDCD10 antagonized the mRNA levels of *IFNB*, *ISG56*, *RANTES*, and *CXCL10*. PDCD10 also suppressed the mRNA expression of *IFNB*, *ISG56*, *RANTES*, and *CXCL10* after poly (I: C) stimulation (Fig. 2F). These results indicated that PDCD10 was an inhibitor of SeV-triggered IFN-β activation.

### PDCD10 deficiency promoted SeV-induced IFN-I production

**Fig 3:**
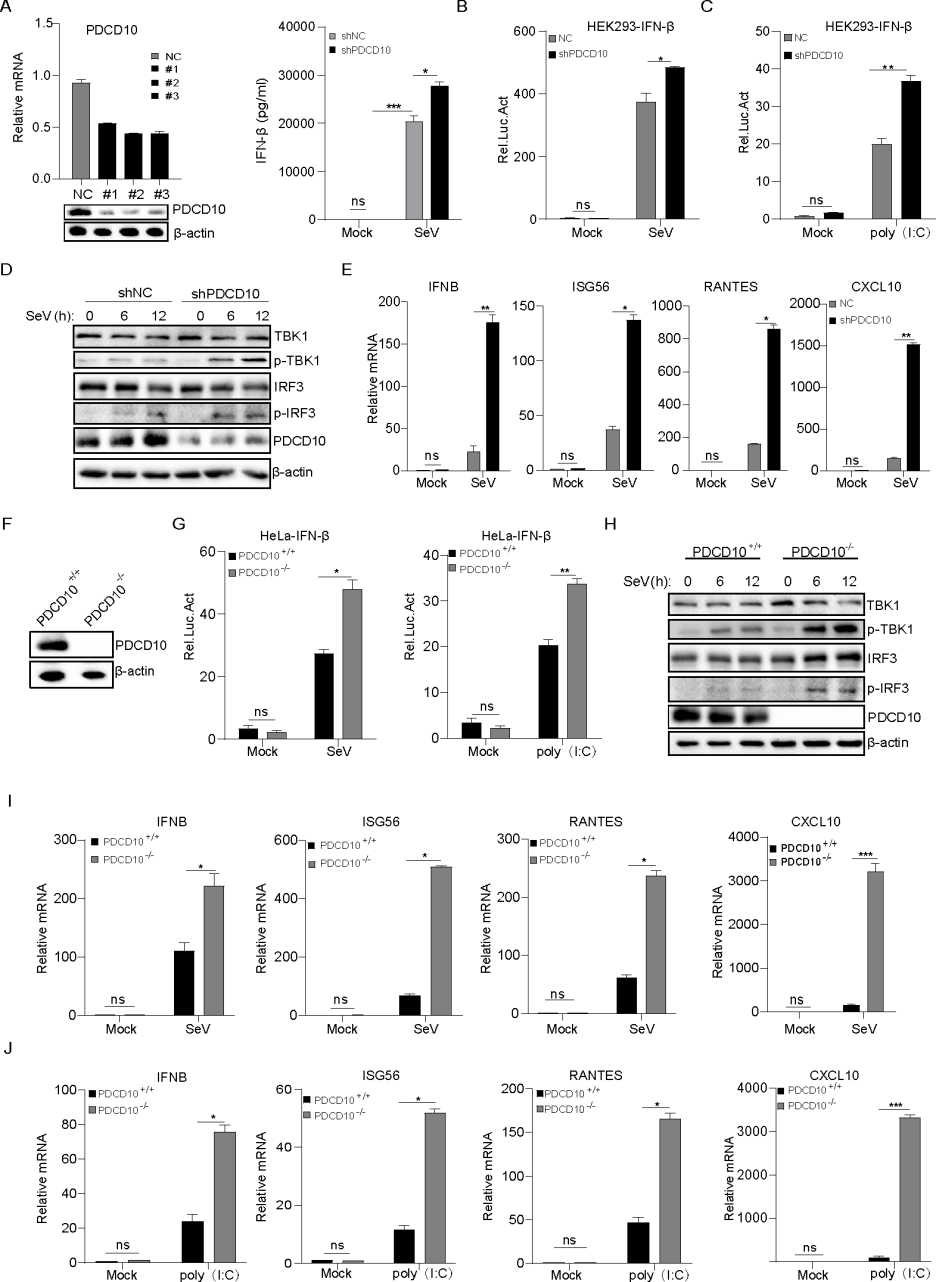
PDCD10 deficiency increased SeV-induced IFN-I activation. (**A**) PDCD10 shRNAs or negative PDCD10 shRNA control (NC) were transfected into HEK293 cells, and after 72 h, the cell lysates were subjected to Western blotting. Negative PDCD10 shRNA control (NC) or different doses of #3 PDCD10 shRNA were transfected into HEK293 cells. After 72 h, cell lysates were subjected to Western blotting. Plasmids of PDCD10 shRNA were transfected into HEK293 cells, and after 72 h, the cells were infected with SeV for 12 h. Then IFN-β was assayed with ELISA. (**B**) Negative PDCD10 shRNA control (NC) or #3 PDCD10 shRNA was transfected into HEK293 cells. After 48 h, cells were transfected with IFN-β-luc, 24 h post-transfection, SeV was infected with the cells for 12 h, and cell lysates were analyzed for the relative intensity of luciferase. (**C**) PDCD10 shRNAs or negative PDCD10 shRNA control (NC) were transfected into HEK293 cells, and after 72 h, poly (I: C) was transfected into the cells for 24 h, and cell lysates were analyzed for the relative intensity of luciferase. (**D**) Negative PDCD10 shRNA control (NC) or #3 PDCD10 shRNA was transfected into HEK293 cells. After 48 h, SeV was infected with the cells for 12 h, and cell lysates were subjected to Western blotting. (**E**) Negative PDCD10 shRNA control (NC) or #3 PDCD10 shRNA was transfected into HEK293 cells. After 48 h, SeV was infected with the cells for 12 h. Cellular RNA was extracted and reverse transcribed into cDNA. Relative mRNA level was detected by qPCR. (**F**) PDCD10 wild-type and PDCD10-knockout HeLa cell lysates were subjected to Western blotting. (**G**) PDCD10-knockout cells were transfected with pRL-TK (20 ng) together with pIFN-β-luc or pISRE-luc (400 ng). After 24 h, the cells were infected with SeV for 12 h or transfected with poly (I: C) (1 µg) for 24 h. Cell lysates were then analyzed for the relative intensity of luciferase. (**H**) PDCD10-knockout cells or PDCD10 wild-type cells were infected with SeV at the indicated time points, and cell lysates were subjected to western blotting assay. (**I and J**) PDCD10-knockout cells were infected with SeV for 12 h, cellular RNA was extracted and reverse transcribed into cDNA. Relative mRNA levels were detected by qPCR. Data are representative of three independent experiments. The data shown are the mean ± s.e.m; * *P* < 0.05, ** *P* < 0.01, *** *P* < 0.001, **** *P* < 0.0001 (two-way ANOVA, GraphPad Prism 8.3.0).

To examine the effect of PDCD10 on SeV-induced innate immune response, we used short hairpin RNAs (shRNA) to knock down PDCD10 (Fig. 3A). #3 PDCD10 shRNA overexpressing HEK293 cells were infected with SeV. ELISA results showed that IFN-β secretion was increased in #3 PDCD10 shRNA expressing cells after SeV or VSV infection (Fig. 3A; Fig. S1C). As shown in Fig. 3B, the results of the luciferase assay showed that compared to PDCD10-negative control (NC) shRNA, IFN-β promoter activation was increased during PDCD10 knockdown after SeV infection. Consistent with this finding, IFN-β promoter activation was enhanced in PDCD10-knockdown HEK293 cells after poly (I: C) stimulation (Fig. 3C). Next, we examined the phosphorylated IRF3 level in PDCD10-knockdown HEK293 cells during SeV infection. The results showed that IRF3 phosphorylation level was increased after PDCD10 knockdown during SeV infection (Fig. 3D). We also noted that the mRNA levels of *IFNB*, *ISG56*, *RANTES*, and *CXCL10* in PDCD10-knockdown cells were increased after viral infection (Fig. 3E).

To further confirm the effect of PDCD10 on innate immune response, we assessed IFN-β promoter activation in PDCD10-knockout cells (PDCD10 KO cells, PDCD10^-/-^) during viral infection. As shown in Fig. 3F, PDCD10 expression could not be detected in PDCD10^-/-^ cells. To verify the IFN-β production in PDCD10^-/-^ cells, luciferase assay, Western blotting, and qPCR were performed. We found that IFN-β promoter activation was increased after SeV infection or poly (I: C) transfection in PDCD10^-/-^ HeLa cells (Fig. 3G). PDCD10 deficiency promoted IRF3 phosphorylation (Fig. 3H; Fig. S1D) and enhanced *IFNB*, *ISG56*, *RANTES*, and *CXCL10* mRNA expression (Fig. 3I; Fig. S1E). PDCD10^-/-^ cells were transfected with poly (I: C), and the results (Fig. 3J) showed that PDCD10 deficiency increased *IFNB*, *ISG56*, *RANTES*, and *CXCL10* mRNA transcription as compared to PDCD10^+/+^ cells (PDCD10 wild-type cells, PDCD10^+/+^). We transfected PDCD10 into PDCD10^-/-^ cells and found that the effect on ISGs mRNA transcription was reversed after poly (I: C) stimulation. Our findings establish that PDCD10 deficiency promoted SeV-induced IFN-β activation.

### PDCD10 interacted and co-localized with VISA

**Fig 4:**
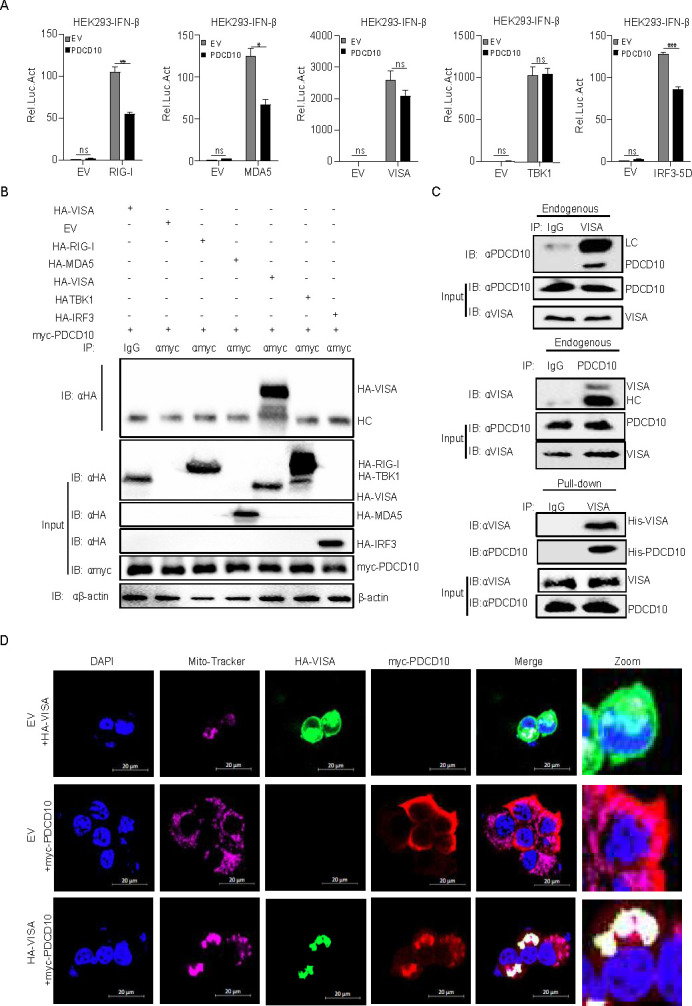
PDCD10 targeted VISA to inhibit IFN-β production. (**A**) Plasmids of PDCD10 (1 µg), pIFN-β-luc (200 ng), and pRL-TK (20 ng) together with RIG-I, MDA5, VISA, TBK1, or IRF3-5D (500 ng) were transfected into HEK293 cells for 24 h, and cell lysates were analyzed for the relative intensity of luciferase. (**B**) PDCD10 was transfected with RIG-I, MDA5, VISA, TBK1, or IRF3; after 24 h, cell lysates were subjected to Co-IP. Anti-HA antibody was used as the IP primary antibody. (**C**) HeLa cell lysates were subjected to Co-IP using the indicated antibodies. Anti-VISA or anti-PDCD10 antibody was used as the IP primary antibody. Meanwhile, PDCD10 and VISA purified proteins were added together, then the samples were subjected to pull-down by using the indicated antibodies. Anti-VISA antibody was used as the IP primary antibody. (**D**) HA-VISA together with EV or myc-PDCD10 was transfected into HeLa cells for 24 h, cells were fixed and subjected to confocal imaging. Data are representative of three independent experiments. The data shown are the mean ± s.e.m; *P < 0.05, **P < 0.01, ***P < 0.001, ****P < 0.0001 (two-way ANOVA, GraphPad Prism 8.3.0).

To investigate the molecular mechanisms by which PDCD10 influences the innate immune response, we investigated whether PDCD10 was involved in RLR component-mediated IFN-I signaling transduction. First, PDCD10 and RLRs signaling components were transfected separately into HEK293 cells, including RIG-I, MDA5, VISA, TBK1, and IRF3-5D (a constantly activating mutant). As shown in Fig. 4A, we found that RIG-I-, MDA5-, and IRF3-5D-induced IFN-β promoter activation was inhibited by PDCD10. However, PDCD10 had no effect on VISA- and TBK1-induced IFN-β production, thus suggesting that PDCD10 may affect IFN-β production at the VISA or TBK1 level. The co-immunoprecipitation assay (Co-IP) and pull-down assay results showed that PDCD10 interacts with VISA (Fig. 4B and C). Because VISA is predominantly located in mitochondria, we determined whether PDCD10 co-localized with VISA. As shown in Fig. 4D, PDCD10 was co-localized with VISA on mitochondria. These results suggested that PDCD10 interacts with VISA to inhibit the innate immune response.

### PDCD10 inhibits IFN-I production by disrupting the VISA-IRF3 complex

**Fig 5:**
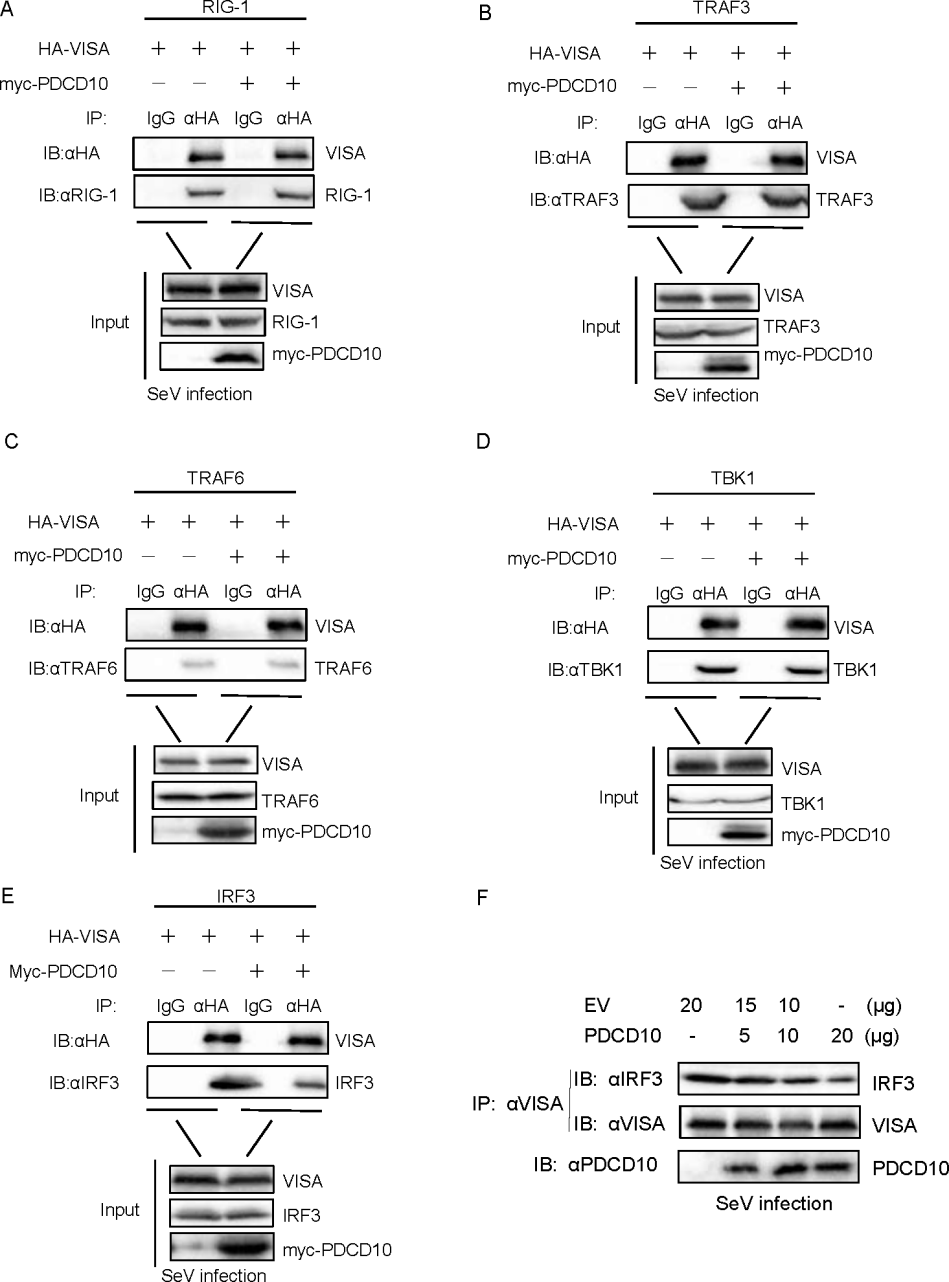
PDCD10 disrupts VISA-IRF3 complex formation after SeV infection. (**A–E**) Plasmids of PDCD10 (10 µg) and VISA (10 µg) together with RIG-I, TRAF3, TRAF6, TBK1, or IRF3 (10 µg) were transfected into HEK293 cells. After 24 h, cell lysates were subjected to Co-IP. (**F**) PDCD10 was transfected into HEK293 cells with different doses. After 24 h, cell lysates were subjected to Co-IP.

Because PDCD10 binds to VISA, to reveal the mechanism by which PDCD10 interacts with VISA to inhibit IFN-β production, we hypothesized that PDCD10 may affect the function of the VISA-associated complex that acts as a signaling transduction platform. For this purpose, VISA, PDCD10, and RIG-I, TRAF3, TRAF6, TBK1, or IRF3 were transfected into HEK293 cells. After 24 h, the cells were infected with SeV and then lysed. Anti-VISA antibody was used as the IP primary antibody, and competitive Co-IP experiments were performed for the indicated proteins. The results indicated that compared to control, PDCD10 expression inhibited VISA-IRF3 interaction, but not RIG-I, TRAF3, TRAF6, or TBK1 complex (Fig. 5A through E). Furthermore, PDCD10 was transfected into HEK293 cells at different doses, as shown in Fig. 5F; PDCD10 inhibited VISA-IRF3 interaction in a dose-dependent manner during SeV infection.

### PDCD10 promotes FMDV replication

**Fig 6:**
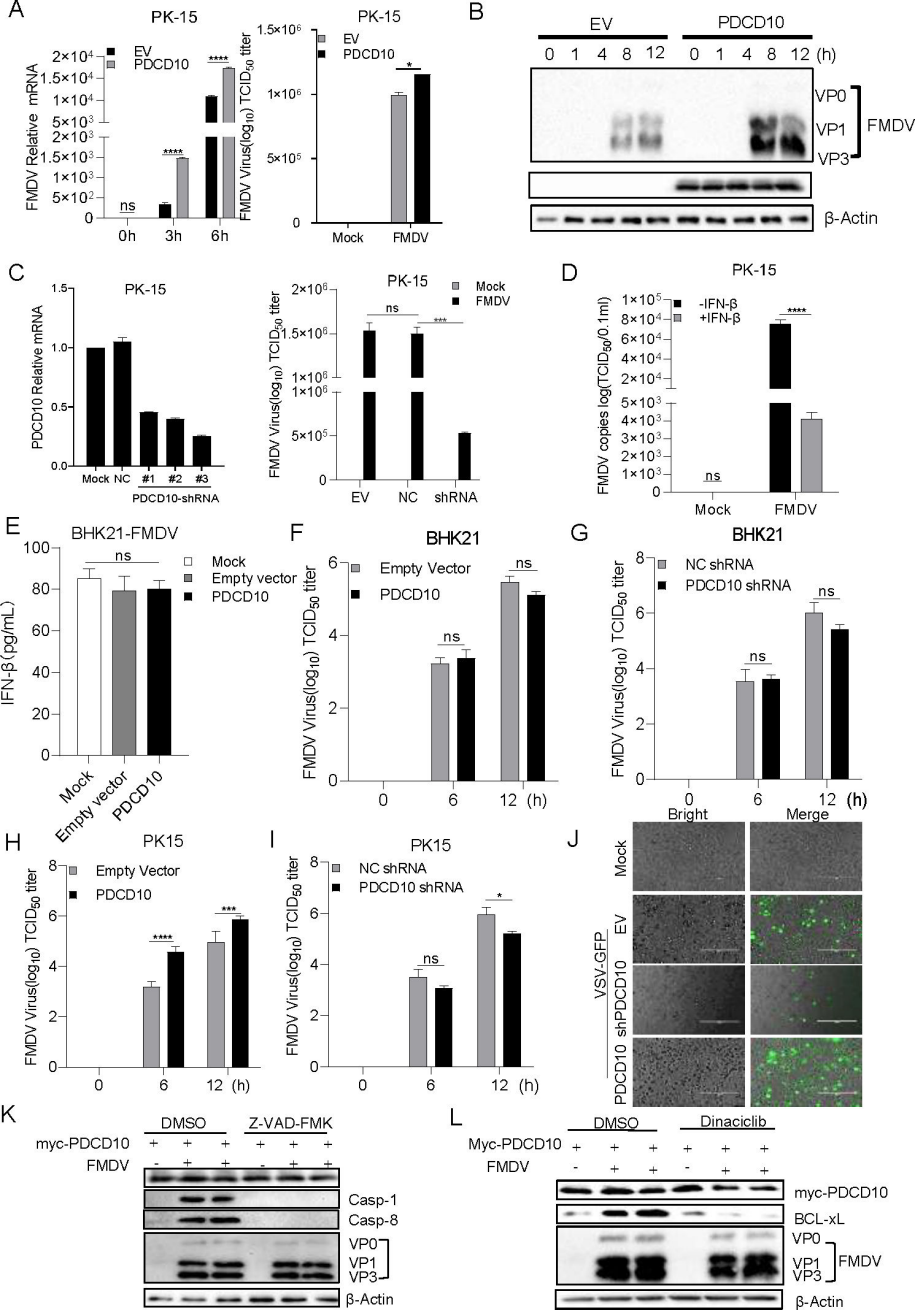
Effect of PDCD10 on FMDV replication. (**A**) PDCD10 or EV (pcDNA 3.1 empty vector, EV) was transfected into PK15 cells. After 24 h, the cells were infected with FMDV (MOI = 5) at the indicated time point. The FMDV titer and relative mRNA level were determined. (**B**) PDCD10 or EV (pcDNA 3.1) was transfected into PK15 cells. After 24 h, the cells were infected with FMDV (multiplicity of infection [MOI] = 5) at the indicated time point, and then cell lysates were subjected to Western blotting. (**C**) PDCD10 shRNA or negative shRNA (NC shRNA) was transfected into PK15 cells, and after 72 h, cells were infected with FMDV at the indicated time points. Cellular RNA was extracted and reverse transcribed into cDNA. The relative mRNA level of PDCD10 and FMDV titer was determined. (**D**) PK15 cells were infected with FMDV; after 3 h, the cells were treated or left untreated with porcine IFNβ. After 6 h, the FMDV titer was detected. (**E**) PDCD10 or EV was transfected into BHK21 cells. After 24 h, the cells were infected with FMDV for 12 h, and the supernatant was collected for the detection of IFN-β by ELISA. (**F and G**) EV, PDCD10, PDCD10-negative shRNA control (NC shRNA), or PDCD10 shRNA was transfected into BHK21 cells. After 24 h or 72 h, the cells were infected with FMDV at the indicated time. Then, FMDV titers were detected. (**H–J**) EV, PDCD10, PDCD10-negative shRNA control (NC shRNA), or PDCD10 shRNA was transfected into PK15 cells. After 24 h or 72 h, the cells were infected with FMDV for the indicated time. The medium supernatant was collected, and UV light was used to inactivate FMDV for 2 h. PK-15 cells were treated with the inactivated FMDV supernatant for 6 h, and the cells were infected with FMDV or VSV-GFP. FMDV titer (**H and I**) and green fluorescence (**J**) were assessed. (**K and L**) PDCD10 was transfected into PK15 cells. After 24 h, Z-VAD-FMK (**K**) or Dinaciclib (**L**) was added, then FMDV was infected with the indicated cells. After 6 h, the cells were lysed and subjected to western blotting detection. Data are representative of three independent experiments. The data shown are the mean ± s.e.m; *P < 0.05, **P < 0.01, ***P < 0.001, ****P < 0.0001 (two-way ANOVA, GraphPad Prism 8.3.0).

Next, we assessed the effect of PDCD10 on FMDV replication. PDCD10 was transfected into PK15 cells, and the FMDV titers were determined. As shown in Fig. 6A, PDCD10 overexpression increased FMDV mRNA expression and viral titer as compared to those of the control. Additionally, as shown in Fig. 6B, the protein levels of VP0, VP1, and VP3 of FMDV were increased after PDCD10 overexpression. PDCD10 shRNA-overexpressing PK15 cells were infected with FMDV, and the results (Fig. 6C) showed that knockdown of PDCD10 decreased FMDV titer. To investigate the effect of IFN-β on FMDV replication, PK15 cells were treated with porcine IFN-β after FMDV infection. The result showed that IFN-β suppressed FMDV replication (Fig. 6D). To confirm whether PDCD10 affected FMDV replication in an IFN-β-dependent manner, PDCD10 was transfected into IFN-β-deficient BHK21 cells. After 24 h, the cells were infected with FMDV for the indicated time period. We observed that the IFN-β secretion level was not affected (Fig. 5E). PDCD10 or PDCD10 shRNA overexpression did not affect the FMDV replication level in BHK21 cells (Fig. 6F and G). In addition, to determine that the promotion of FMDV replication by PDCD10 correlates with the inhibition of IFNβ production, PK15 cells were transfected with PDCD10, and the cells were then infected with FMDV for 12 h. Subsequently, the medium was collected, and UV light was used to inactivate the FMDV. PK15 cells were pre-treated with the inactivated FMDV medium, and the FMDV titer was assayed. The results showed that in IFN-producing normal PK-15 cells, PDCD10 promoted FMDV replication after treatment with the FMDV inactivated supernatant as compared to control (Fig. 6H). Additionally, PDCD10 knockdown inhibited FMDV replication as compared to EV control after treatment with the inactivated FMDV supernatant (Fig. 6I).

To assess the effect of PDCD10 on antiviral status, PK15 cells were transfected with PDCD10, and the cells were then infected with FMDV for 12 h. Subsequently, the medium was collected, and UV light was used to inactivate the FMDV. PK15 cells were pre-treated with the inactivated FMDV medium, and the cells were then infected with VSV-GFP. After the indicated time period, VSV-GFP was detected. As shown in Fig. 6J, the fluorescence of VSV-GFP increased after PDCD10 expression. Conversely, PDCD10 knockdown decreased the fluorescence intensity of VSV-GFP. Consistently, PDCD10 knockout cells were treated with VSV-RFP (containing the red fluorescent protein), and fluorescence of VSV-GFP increased after PDCD10 knockout (Fig. S1F). Moreover, previous studies have demonstrated that PDCD10 is associated with apoptosis and the cell cycle. To clarify whether PDCD10 may promote viral replication by regulating apoptosis or the cell cycle, Z-VAD-FMK (apoptosis inhibitor) or Dinaciclib (cell cycle inhibitor) was added in PDCD10-overexpressing cells during FMDV infection. In the results shown in Fig. 6K and L, Z-VAD-FMK and Dinaciclib did not have an effect on FMDV replication during PDCD10 expression. These results suggested that PDCD10-inhibited IFN-β production may affect FMDV replication.

### FMDV 3A cooperated with PDCD10 to inhibit I-IFN production

**Fig 7:**
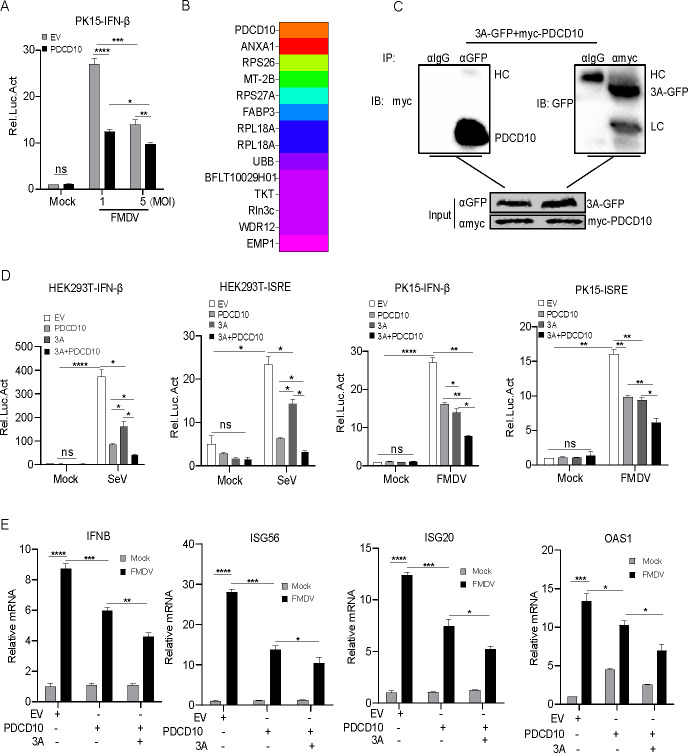
FMDV 3A cooperates with PDCD10 to inhibit IFN-I production. (**A**) PDCD10 (1 µg), pRL-TK (40 ng), and pIFN-β-luc (400 ng) plasmids were transfected into PK-15 cells. After 24 h, cells were treated or untreated with FMDV (multiplicity of infection [MOI] = 1 or 5). After 12 h, cell lysates were analyzed for the relative intensity of luciferase. (**B**) FMDV-encoded proteins were transfected into PK15 cells, separately. After 24 h, cell lysates were subjected to immunoprecipitation mass spectrometry detection. (**C**) HEK293 cells were transfected with PDCD10 and FMDV 3A protein. After 24 h, cell lysates were subjected to IP detection. Anti-Flag or anti-GFP was used as the IP primary antibody. (**D**) Plasmids of EV (400 ng), PDCD10 (300 ng) plus EV (100 ng), or PDCD10 (300 ng) plus 3A (100 ng), and pRL-TK (20 ng) together with pIFN-β-luc or pISRE-luc (200 ng) were transfected into HEK293 or PK15 cells. After 24 h, the cells were infected with SeV or FMDV for 12 h, and cell lysates were analyzed for the relative intensity of luciferase. (**E**) Plasmids of EV (1 µg), PDCD10 (500 ng) plus EV (500 ng), or PDCD10 plus 3A (500 ng) were transfected into PK15 cells. After 24 h, the cells were infected with FMDV (MOI = 5) for 9 h. Cellular RNA was extracted and reverse transcribed into cDNA. Relative mRNA level was detected by qPCR. Data are representative of three independent experiments. Data are representative of three independent experiments. The data shown are the mean ± s.e.m; *P < 0.05, **P < 0.01, ***P < 0.001, ****P < 0.0001 (two-way ANOVA, GraphPad Prism 8.3.0).

The effect of FMDV on PDCD10-suppressed IFN-β production was assessed. As shown in Fig. 7A, compared to EV, PDCD10 inhibited FMDV-induced IFN-β promoter activation as determined by luciferase detection. We used low virus titer and high virus titer to investigate the effect of FMDV on PDCD10-mediated innate immune response. We found that using a high titer of FMDV strengthens PDCD10-mediated inhibition of IFN-β promoter activation (Fig. 7A). This finding suggested that FMDV components might be involved in PDCD10-mediated IFNβ inhibition.

To investigate the involvement of FMDV components in PDCD10-mediated innate immune response, different FMDV proteins were separately transfected into PK15 cells, and immunoprecipitation mass spectrometry was performed. The data showed that PDCD10 interacted with 3A (Fig. 7B). To further confirm that 3A interacted with PDCD10, 3A and PDCD10 were transfected into HEK293 cells. We performed a Co-IP assay and observed that 3A interacted with PDCD10 (Fig. 7C). In addition, compared to PDCD10 expression alone, we found that the combination of 3A and PDCD10 showed a higher inhibition effect on IFN-β promoter activation and the mRNA transcription expression of *IFNB*, *ISG54*, *ISG20*, and *OAS1* (Fig. 7D and E).

Taken together, the above-mentioned results suggested that 3A cooperated with PDCD10 to inhibit IFN-I production.

### Full length of FMDV 3A interacted with PDCD10 to promote FMDV replication

**Fig 8:**
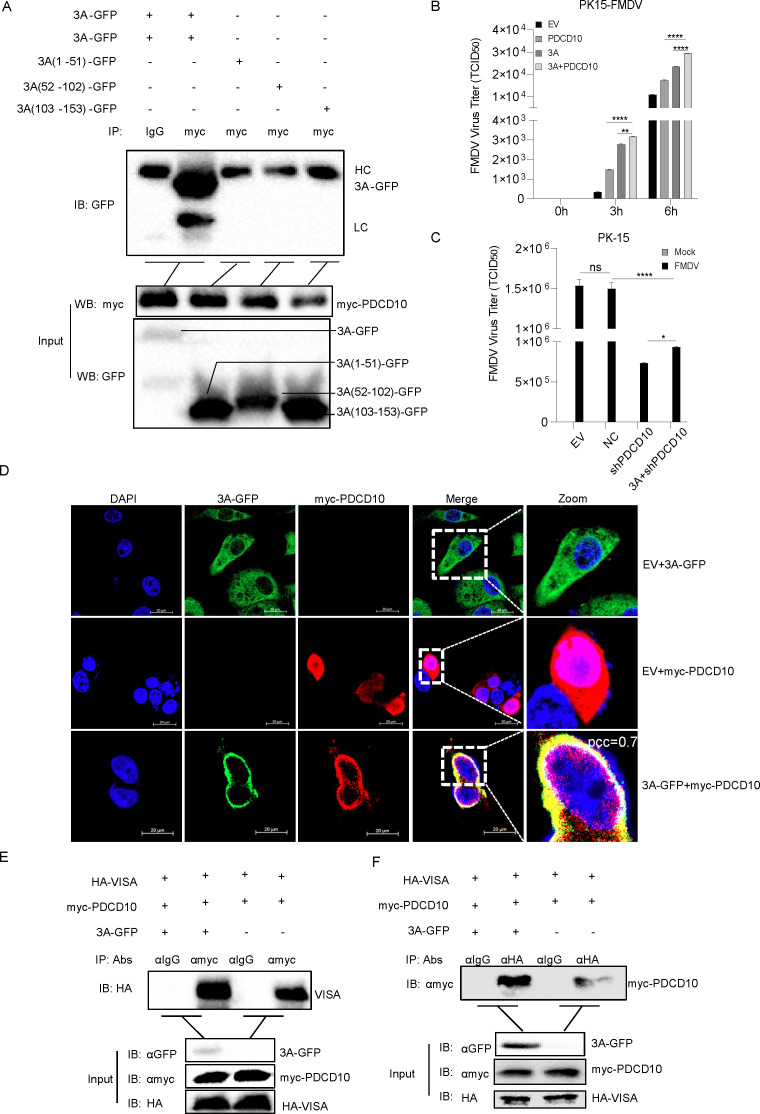
Full-length FMDV 3A promotes PDCD10-VISA complex formation. (**A**) Plasmids of PDCD10 together with full-length 3A or its mutants with a GFP tag were transfected into HEK293 cells. After 24 h, cell lysates were subjected to Co-IP, and anti-myc monoclonal antibody was used as the IP primary antibody. (**B**) Plasmids of FMDV 3A, PDCD10, EV, or 3A plus PDCD10 were transfected into PK15 cells. After FMDV infected the cells at the indicated time point, the supernatant was subjected to FMDV titer detection. (**C**) Plasmids of FMDV 3A, PDCD10 shRNA, NC, or 3A plus PDCD10 shRNA were transfected into PK15 cells. After FMDV infected the cells for 6 h, the cells were subjected to FMDV titer detection. (**D**) Plasmids of myc-PDCD10 and 3A-GFP were transfected into HeLa cells. After 24 h, the cells were fixed and subjected to confocal image observation. (**E and F**) Plasmids of 3A-GFP or EV together with VISA and PDCD10 were transfected into HEK293 cells. After 24 h, the cell lysates were subjected to Co-IP. Data are representative of three independent experiments. The data shown are the mean ± s.e.m; *P < 0.05, **P < 0.01, ***P < 0.001, ****P < 0.0001 (two-way ANOVA, GraphPad Prism 8.3.0).

We co-expressed 3A deletion mutants with PDCD10 to determine the 3A domains responsible for PDCD10 binding. As shown in Fig. 8A, the full-length 3A protein bound to PDCD10, thus suggesting that PDCD10 may interact with the conformational structure of 3A. PK15 cells were transfected separately with 3A, PDCD10, or 3A plus PDCD10. After 24 h, PK15 cells were infected with FMDV. We noted that the promoting effect of 3A plus PDCD10 on FMDV replication was more intensive than 3A or PDCD10 expression alone (Fig. 8B). Next, PK15 cells were transfected with PDCD10 shRNA and 3A and then infected with FMDV. Compared to PDCD10-negative shRNA control, the FMDV titer was decreased after 3A transfection in PDCD10-interfering cells (Fig. 8C). Moreover, co-localization assays were performed to confirm the association between 3A and PDCD10. HeLa cells were transfected with 3A-GFP and PDCD10. As shown in Fig. 8D, either 3A or PDCD10 localized to the cytoplasm and plasma membrane with some highly concentrated spots around the nucleus. The distribution of 3A overlapped with that of PDCD10 (pcc = 0.789). To investigate the mechanism by which 3A cooperated with PDCD10 to promote FMDV, 3A, PDCD10, and VISA were transfected into HEK293 cells, and co-IP results showed that 3A promoted the formation of the VISA-PDCD10 complex (Fig. 8E and F). These results suggested that FMDV replication was promoted by interaction between the full length of 3A protein and PDCD10.

## DISCUSSION

FMDV infection triggers host innate immune response, such as production of IFNs and induction of inflammatory cytokines (20–22). An increase in IFN-β expression is one of the important approaches to defend against foreign viral infection. RLRs-mediated IFN-β induction is well investigated.

FMDV has a single positive-strand RNA genome. The RNA genome of the virus is recognized by RLRs, which bind to VISA to recruit TBK1. Eventually, the phosphorylated TBK1 activates IRF3 to translocate into the nucleus and induce IFN-I production (23). In the present study, we demonstrated that PDCD10 acted as a candidate repressor for SeV-induced RLR signaling transduction. PDCD10 belongs to the *PDCD* gene family, which is ubiquitously expressed and conserved (9). In the past decade, several studies have shown that PDCD10 acts as an essential regulator in vasculogenesis, autophagy, and apoptosis (7, 24, 25). Recent studies have revealed that PDCD10 is associated with immune response, such as B-cell depletion halting the maturation and progression of already formed ectatic blood vessels into multicavernous clinically significant lesions in PDCD10^+/−^ murine models (26). PDCD10 also has a regulatory effect on other immune-related genes to improve immune response and suppress the development of inflammation according to the Bayesian gene regulatory network (27). Innate immune response is the first line of defense against foreign pathogens; however, information regarding the influence of PDCD10 on immune responses remains limited. Our present study is the first to identify that PDCD10 altered RNA virus-induced IFN-β production to promote FMDV replication.

By using PDCD10 knockdown and knockout models, we demonstrated that PDCD10 negatively regulated RNA virus-induced host antiviral defense. Mechanistically, through direct interaction with VISA, PDCD10 blocked VISA-mediated RLRs signaling transduction. VISA is a central platform for RNA nucleic acid sensing. We found that PDCD10 inhibited the formation of the VISA-IRF3 complex. Extensive studies have shown that host proteins play a role in strictly regulating RLRs signaling transduction, for example, lactate directly interacts with VISA trans-membrane domain (TM) to prevent its aggregation (28); UBXN1 interacts with VISA to disrupt its oligomerization and the MAVS-TRAF3-TRAF6 complex formation during viral infection (29). VISA acts as an adapter for activating distinct signaling pathways that lead to IRF3 and NF-κB activation. Siqi Liu et al. found that during virus infection, the phosphorylated MAVS binds to IRF3 and recruits IRF3 for its phosphorylation and activation (30). VISA is associated with TRAF motifs of TRAF2 and TRAF6 to facilitate RIP and NEMO K63-linked poly-ubiquitination, which leads to NF-κB activation (31, 32). VISA interacts with TRAF3, which is essential in virus-induced IRF3 activation (33, 34). Our results showed that PDCD10 did not disrupt VISA-TRAF6 interaction; thus, we speculated that PDCD10 might not affect the NF-κB signaling transduction during the RNA virus infection. Phosphorylated VISA interacts with IRF3 to induce IFNβ activation (30). In our present study, we found that PDCD10 inhibited the formation of VISA-IRF3 complex after SeV infection; however, the underlying mechanism requires further research. A previous study has shown that MST1 was associated with TBK1 and IRF3 to impede IRF3 phosphorylation to shut off the antiviral response of the RIG-I−MAVS-IRF3 axis (35). Several studies have shown that in the multicomponent complexes of STRIPAK, the STRN family proteins (STRNs) share a conserved PDCD10-binding region to directly interact with PDCD10, which acts as a protein bridge recruiting GCKIII kinases to STRNs (36–39). GCKII kinases MST1/2 are associated with IRF3 activation (35). Therefore, we speculated that PDCD10 might affect VISA-IRF3 antiviral response through STRIPAK. This, however, is only a hypothesis that requires further confirmation for the precise mechanism.

Next, we attempted to discover the mechanism by which FMDV replication is promoted by PDCD10. PDCD10 was overexpressed in IFN-β-deficient BHK21 cells, and the cells were infected with FMDV. We observed that the FMDV titer in the PDCD10-transfected cells did not increase as compared to EV. These results were further confirmed in PDCD10-knockdown BHK21 cells; this suggested that PDCD10 may promote the replication of FMDV by affecting IFN-β production. These results reinforced the hypothesis that PDCD10 was involved in viral-induced innate immune response.

In the process of evolution, viruses may take advantage of host proteins to counter the innate immune response, thereby creating a favorable environment for their replication. For example, H7N9 PB1-F2 induces lysosomal and proteasomal degradation of aggregated VISA (40); the NS1 protein of influenza A viruses targets TRIM25 to prevent ubiquitination of RIG-I, which blocks IFN production (41). FMDV 3A also cooperates with DDX56 to inhibit IRF3 phosphorylation (4). The 3A protein of FMDV plays a critical role in constructing the viral replication complex, which is important for replication. However, the precise mechanisms by which 3A affects FMDV replication remain incompletely understood. In the present study, we found that the full length of the 3A protein interacted and co-localized with PDCD10 to reduce FMDV-induced IFN-I production. A previous study showed that 3A anchors to intracellular membranes to form the replication complex (42). Hence, we speculated that 3A may also anchor to intracellular membranes, such as the mitochondrial membrane, together with PDCD10 to potentiate the inhibition of VISA-mediated innate immune response; further studies are required to confirm this speculation. A previous study showed that FMDV 3A also interacts with VISA (43). In the present study, we found that PDCD10 was associated with 3A and VISA; thus, we speculated that PDCD10-3A, together with VISA, formed a complex to inhibit VISA signaling transduction. This requires further study for confirmation.

**Fig 9:**
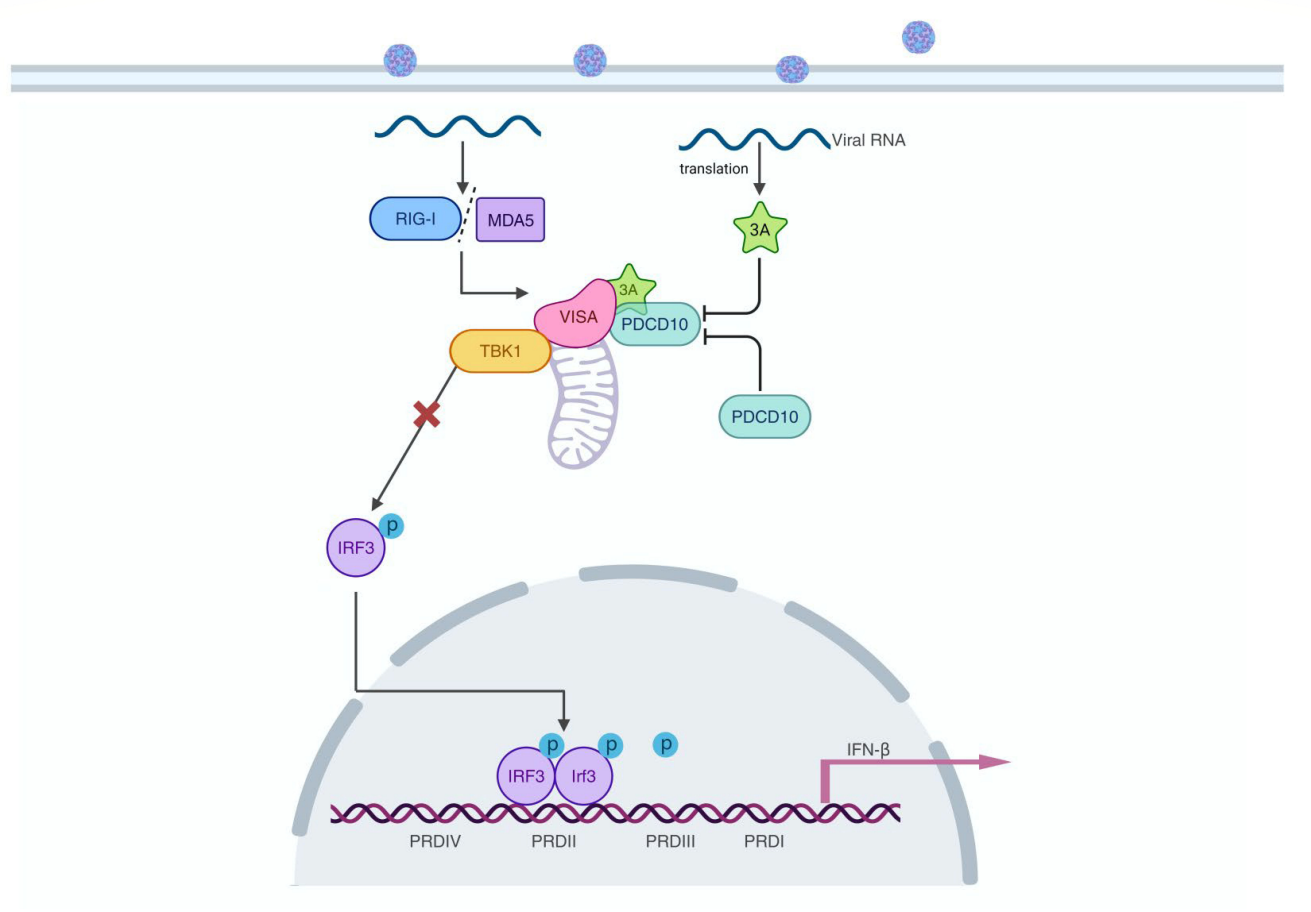
Model of FMDV 3A cooperates with PDCD10 to inhibit IFN-I production to promote FMDV replication. FMDV infection increased PDCD10 expression levels, and PDCD10 interacts with VISA to disrupt the formation of the VISA-IRF3 complex that suppresses IFN-I production. The FMDV 3A protein cooperates with PDCD10 to promote PDCD10-VISA complex formation and inhibits IFN-I production to promote FMDV replication.

Collectively, our results showed that after SeV infection, PDCD10 bound to VISA to disrupt the formation of VISA-IRF3 complex, which suppressed IFN-β activation; PDCD10 also cooperated with the 3A protein to promote FMDV replication (Fig. 9). This study provided new insights into the mechanism by which PDCD10 affected viral-induced IFN-β activation and revealed that FMDV 3A cooperated with PDCD10 to strengthen the inhibition effect on IFN-I production.

## Data Availability

The authors confirm that the data supporting the findings of this study are available within the article.

## SUPPLEMENTAL MATERIAL

Fig. S1 jvi.00657-25-s0001.tif PDCD10 inhibits VSV-induced IFN-I production.

Supplemental legend jvi.00657-25-s0002.docx Legend for Fig. S1.

ASM does not own the copyrights to Supplemental Material that may be linked to, or accessed through, an article. The authors have granted ASM a non-exclusive, world-wide license to publish the Supplemental Material files. Please contact the corresponding author directly for reuse.
