# Supplementary material for: FMDV 3A cooperates with PDCD10 to promote FMDV replication by inhibiting VISA-mediated innate immunity
Source: J Virol. 2025 Nov 26;99(12):e00657-25. doi: 10.1128/jvi.00657-25 (PMC12724370; doi:10.1128/jvi.00657-25)
Supplement: Supplemental legend — Legend for Fig. S1. [file jvi.00657-25-s0002.docx]

**Figure S1. PDCD10 deficiency increased VSV induced IFN-I activation and overexpression suppressed VSV induced IFN-I activation.**

**(A)** Plasmids of PDCD10 was transfected into HEK293 cells, after 24 h, the cells were infected with VSV for 12 h, then IFN-β was assayed with ELISA. **(B)** PDCD10 plasmid (1 μg) was transfected into HEK293 cells. After 24 h, the cells were infected with VSV for 12 h, cell RNA was extracted and reverse transcribed into cDNA. Relative mRNA levels were detected by qPCR. **(C)** Plasmids of PDCD10 shRNA was transfected into HEK293 cells, after 72 h, the cells were infected with VSV for 12 h. then IFN-β was assayed with ELISA. **(D)** PDCD10-knockout cells or PDCD10 wild-type cells (HEK293) were infected with VSV at the indicated time points, and cell lysates were subjected to western blotting assay. **(E)** PDCD10- wildtype or knockout cells were infected with VSV for 12 h, cells RNA was extracted and reverse transcribed into cDNA. Relative mRNA levels were detected by qPCR. **(F)** PDCD10-knockout cells or PDCD10 wild-type cells were infected with VSV-RFP, and red fluorescence were assessed. Data are representative of three independent experiments. The data shown are the mean±s.e.m; * *p*<0.05, ** *p*<0.01, *** *p*<0.001, **** *p*<0.0001 (two-way ANOVA, Graphpad prism 8.3.0).
